# ﻿Taxonomic notes on the genus *Deutzia* (Hydrangeaceae) from Central China

**DOI:** 10.3897/phytokeys.220.96623

**Published:** 2023-02-24

**Authors:** Song-Zhi Xu, Qi-Liang Gan, Zhen-Yu Li

**Affiliations:** 1 School of Life Science, Nantong University, Nantong, Jiangsu 226019, China Nantong University Nantong China; 2 Zhuxi Qiliang Biological Institute, Zhuxi, Hubei 442300, China Zhuxi Qiliang Biological Institute Hubei China; 3 State Key Laboratory of Systematic and Evolutionary Botany, Institute of Botany, Chinese Academy of Sciences, Beijing 100093, China Institute of Botany, Chinese Academy of Sciences Beijing China

**Keywords:** *Deutziasetchuenensis*, lectotype, new variety, var. *macrocarpa*

## Abstract

Based on examination of syntype specimens deposited at P, the lectotype for the name *Deutziasetchuenensis* Franch. is designated here. By consulting literature and specimen records, the type locality of D.setchuenensisvar.longidentata Rehder, ‘Chin-Ting shan’ in the protologue is likely a misspelling of ‘Chiuting shan’ which is now called Jiuding shan located in southern Mao county, Sichuan province. In addition, a new variety, Deutziasetchuenensisvar.macrocarpa Q.L.Gan, Z.Y.Li & S.Z.Xu from western Hubei, Central China, is described and illustrated. It differs from other varieties of *D.setchuenensis* Franch. by the orange anthers, broader outer filaments, obtuse inner filaments, and larger fruits.

## ﻿Introduction

*Deutzia* Thunb. as the second largest genus of the tribe Philadelpheae (Hydrangeaceae), consists of ca. 60 species and is mainly distributed across eastern Asia and Mexico, with ca. 50 species found in China (Hwang et al. 2001; Hufford 2004). *Deutziasetchuenensis* Franch. and its varieties, which were native to China, were widely introduced as ornamental plants (Hutchinson 1909; Rehder 1940; Brickell and Zuk 1996). Recently, when we were identifying *Deutzia* specimens from Zhuxi county, Hubei province, we found a wild *Deutzia* morphologically similar to *Deutziasetchuenensis*, but with larger capsules. In order to identify this taxon, we have consulted protologue and specimens. However, we found that the type of *D.setchuenensis* is not designated, evidenced by the fact that all three gatherings (MNHN-P-P04573103, MNHN-P-P04573105 & MNHN-P-P04573106) stored at Muséum d’Histoire Naturelle, Paris (P) (Thiers 2016) were referred as the type. This necessitated designation of a single specimen as the type of this species from the aforesaid syntype. After examinations of syntypes at P, the lectotype of the species is designated in this study. In addition, we found the type locality of D.setchuenensisvar.longidentata Rehder remains confused, and ‘Chin-Ting shan’ in the original record is likely a misspelling for Chiuting shan (now Jiuding shan). Finally, after checking Flora of China (Hwang et al. 2001), and relative literature and making comparisons with the specimens of *Deutzia* stored in PE and some virtual specimen databases (P, A, CVH, and JSTOR), we found that this unknown taxon resembles *Deutziasetchuenensis* in stem, leaf, flower, fruit and indumentum, white disc, and smaller seeds, but differs from three varieties of *D.setchuenensis* in the color of anthers, shape of outer and inner stamens, and size of fruits (Hutchinson 1909; Rehder 1911; Hwang 1992, 1995; Hwang et al. 2001). Therefore, we confirm that these peculiar plants represent a new variety of *Deutziasetchuenensis*, which is described and illustrated here.

## ﻿Results

### ﻿Lectotypification of *Deutziasetchuenensis* Franch.

#### ﻿ Deutzia setchuenensis

Taxon classificationPlantaeCornalesHydrangeaceae

Franch. in Journ. De Bot. (Morot) 10: 282. 1896

65E0FCA0-6960-543A-9C76-F343EF0C42F2

##### Type.

China. ‘Set-Chuen orientalis, circa Tchen-kéou-tin’ (eastern Sichuan, near Chengkou tin), P. Farges s.n. (lectotype, P, P04573103 designated here; isolectotypes, P, P04573105 & P04573106; photos PE!).

##### Note.

Adrien René Franchet (1896) published the species based on the type collected from Chengkou tin by P. Farges. There are three specimens collected from Chengkou tin by Farges deposited in Muséum d’Histoire Naturelle, Paris (P), and the label data of the specimens were exactly the same as original records, including the collector, collection locality, and all of them were flowering branches. Of them, two specimens (P04573103 and P04573105) were determined by Franchet, while another (P04573106) was determined by Alfred Rehder. It is clear that these three specimens are the syntypes of *Deutziasetchuenensis*. Based on examination of the syntypes, we selected the more perfect one (P04573103) as lectotype for the species. French missionary and plant collector, Paul Guillaume Farges collected more than 4000 specimens in Chengkou tin from 1892 to 1896 (Bretschneider 1898; Cox 1945). Chengkou tin (1822–1912) was previously located in the administrative division of Qing dynasty, an area renamed Chengkou county since 1913.

### ﻿Correction of the type locality

#### ﻿ Deutzia setchuenensis var. longidentata

Taxon classificationPlantaeCornalesHydrangeaceae

Rehder in Sargent, Pl. Wils. 1: 8. 1911.

95081300-5A69-5C04-B1D9-FF372FE23837

##### Type.

China. western Szechuan (Sichuan): Chiuting shan (original record misspelled it as Chinting), thickets, alt. 1200–1500 m, 25 May 1908, E.H. Wilson 2895 (holotype, A, A0042097; photo, PE!).

##### Note.

According to ‘PlantaeWilsonianae’, from late spring to summer, 1908, E.H. Wilson collected plant specimens along the Min River valleys, and in late May when he was in Jiuding shan (Chiuting shan) around 31°51'N, 103°76'E, southern Mao county, not Jinding (Chinting), the main peak of Emei mountain (29°52'N, 103°33'E).

### ﻿Taxonomic treatment of new variety

#### ﻿ Deutzia setchuenensis Franch. var. macrocarpa

Taxon classificationPlantaeCornalesHydrangeaceae

Q.L.Gan, Z.Y.Li & S.Z.Xu var. nov.

1E45F5A7-063C-5C17-9275-144D3E6A7859

urn:lsid:ipni.org:names:77314712-1

Figs 1 , 2

##### Diagnosis.

The new variety, Deutziasetchuenensis Franch. var. macrocarpa Q.L.Gan, Z.Y.Li & S.Z.Xu can be easily distinguished from other varieties ( var. setchuenensis , var. corymbiflora (Lemoine) Rehder, var. setchuenensis and var. longidentata Rehder) by its orange anthers (vs. yellow anthers), broadly oblong outer filaments with 2 small repand denticles at apex, the width of teeth is more than twice its length (vs. oblong, with 2 deltoid, oblong or lanceolate teeth at apex, the length of teeth is equal to or more than its width), obtuse apex of inner filaments (vs. 2-dentata at apex), and larger fruits 5–7 mm in diam. (vs. 4–5 mm in diam.).

**Figure 1.**
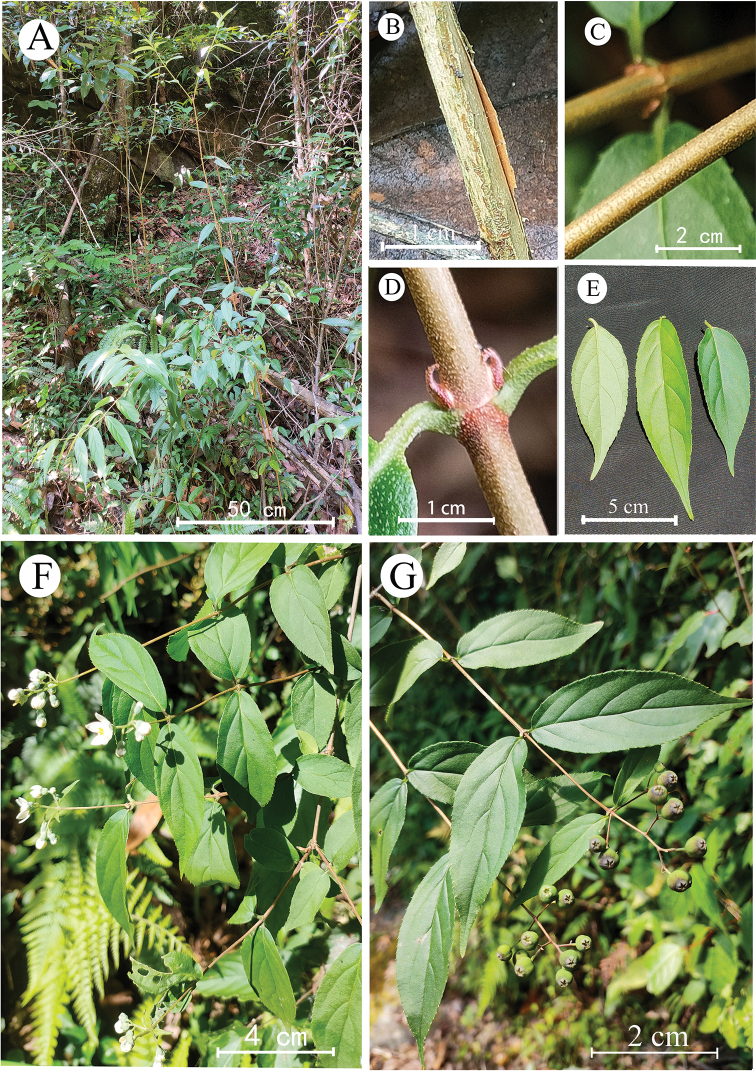
Deutziasetchuenensisvar.macrocarpa, var. nov. **A** plant **B, C** branches **D** petioles **E** leaves **F** flowering branches **G** fruiting branches.

##### Type.

China. Hubei Province: Hongyangou village, Quanxi town, Zhuxi county, alt. 850m, 25 June 2022, Q.L.Gan 3306 (fl., holotype, PE!; isotype, PE!).

##### Paratypes.

China. Hubei province: Hengduanshan, Baguashan Natural Reserve, Zhuxi county, alt. 840m, 13 June 2022, Q.L.Gan 3305 (fl., PE!); the same locality, 2 August 2022, Q.L.Gan 3307 (fr., PE!).

##### Description.

Deciduous shrubs, 90–150 cm tall. Old stems pale gray-brown, often with flaky bark; branches erect to spreading; branchlet opposite, sparsely gray stellate pubescent; flowering branchlets 8–14 cm, 6–10-leaved. Leaves opposite, stipules absent; petiole 3–5 mm long, sparsely stellate-pubescent; leaf blades papery, ovate to ovate-lanceolate, 2–10 cm long, 0.6–3 cm wide, base rounded to broadly cuneate, margin serrulate, apex acuminate or caudate-acuminate, adaxial surface green, not gloss, sparsely stellate pubescent, trichomes 2–4-rayed, abaxial light green, trichomes 4–6-rayed; lateral veins 2–4 paired, mid-vein and lateral veins impressed abaxially, and slightly prominent adaxially, veinlets inconspicuous. Cymes 2–3.5 cm long, 2–3 cm across, 6–12-flowered, sparsely stellate-pubescent; peduncle slender; pedicels 3–6 mm long, usually with 1 to 2 bracts at base or around the middle; bracts linear, 3–6 mm long, 0.5–1 mm wide; flower buds spheroical. Hypanthium hemispheric, 2.5–3.5 mm long and wide, densely 10–13-rayed stellate-tomentulose; calyx lobes 5, broadly deltoid, 1–1.5 × ca. 2 mm, apex acute, erect in bud, spreading in anthesis, inflexed and persistent in fruit. Corolla pure white; petals 5, white stellate-pubescent outside. Stamens 10 in 2-series, erect, filaments pure white, dorsiventraly flattened, anthers orange; outer stamens 4–5 mm long, filaments broadly oblong, with 2-repand denticles at apex, the width of teeth is more than twice its length, anthers broadly ovate; inner stamens shorter than outer ones, filaments obtuse at apex, anthers borne near middle of filaments abaxially, the width of anther exceeds the length. Disc annular, flattened, white. Ovary inferior, 2–3-loculed; styles 2–3, 3–3.5 mm long, usually coherent, glabrous. Capsule subglobose, 5–7 mm in diam., densely stellate-tomentulose, 2–3-valved. Seeds numerous, dark brown, ellipsoid or ovoid, 0.6–0.8 mm long, reticulate.

**Figure 2.**
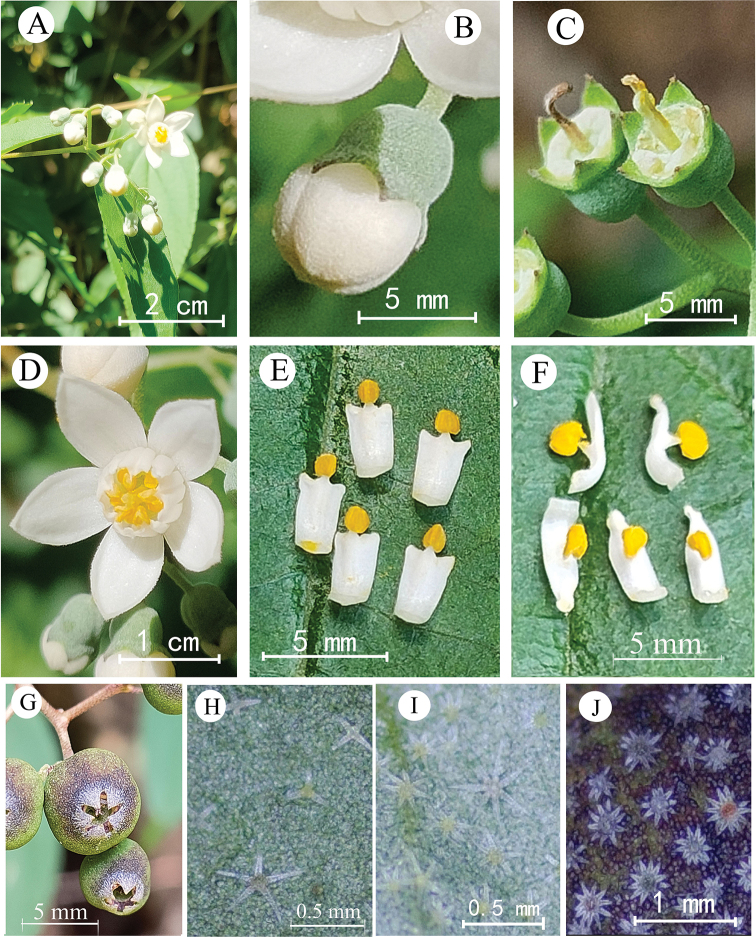
Deutziasetchuenensisvar.macrocarpa, var. nov. **A** flowering branch **B** flower bud **C** young fruit **D** flower **E** outer stamens **F** inner stamens **G** capsules **H** stellate-pubescent on adaxial surface of leaf blade **I** stellate-pubescent on abaxial surface of leaf blade **J** stellate-tomentulose on surface of capsules.

##### Phenology.

Flowering from May to June; fruiting from September to October.

##### Distribution and habitat.

Deutziasetchuenensisvar.macrocarpa distributes in Quanxi town (Hongyangou village), Baguashan Provincial Nature Protection Area, and Taoyuan town (Hetaoyuan village) of Zhuxi county, Hubei province. It occurs at the edge of sparse thickets or forests on hillsides, or by streams. The main companion species include trees: *Phoebezhennan* S. Lee, *Sycopsissinensis* Oliv. and *Symplocoslucida* (Thunb.) Sieb. & Zucc.; shrubs: *Rubusswinhoei* Hance and *Camelliacuspidata* (Kochs) Wright ex Gard.; vines: *Smilaxglaucochina* Warb. and *Actinidiapolygama* (Sieb. et Zucc.) Maxim.; and a fern such as *Dryopterisfuscipes* C. Chr.

##### Etymology.

The Latin name of the variety, ‘macrocarpa’, refers to the large fruit.

##### Vernacular name.

Da Guo Sou Shu (Chinese).

##### Conservation assessment.

Deutziasetchuenensisvar.macrocarpa is currently known only from three localities consisting of less than 20 individuals in Zhuxi county, Hubei province. The provisional conservation status is Critically Endangered (CR), based on criterion D (number of mature individuals fewer than 50) (IUCN 2022).

##### Economic uses.

*Deutziasetchuenensis* has rich intraspecific and morphological genetic diversity (Table 1). In the late 19^th^ century, Deutziasetchuenensisvar.setchuenensis and var. corymbiflora (Lemoine) Rehder were introduced into western Europe, and it was found that the ornamental value and winter hardness of the former were inferior to the latter. The new variety has larger flowers and fruits, and utilization of its germplasm is potential.

**Table 1.** Morphological comparisons of four varieties of *Deutziasetchuenensis* Franch.

Characters	var. setchuenensis	var. longidentata Rehder	var. corymbiflora (E. Lemoine ex Andre) Rehder	var. macrocarpa Q.L.Gan, Z.Y.Li & S.Z.Xu
Inflorescence	6–12-flowered	6–12-flowered	12–50 (or more)-flowered	6–12-flowered
Teeth of outer filaments	oblong, slightly longer than the anthers	lanceolate, much longer than the anthers	deltoid, ca. as long as the anthers	repand, much shorter than the anthers
Apex of inner filaments	2-dentate	2-dentate	2-dentate	obtuse
Anthers	yellow	yellow	yellow	orange
Fruits	4–5 mm in diam.	ca. 4 mm in diam.	ca. 4 mm in diam.	5–7 mm in diam.
Distribution	Chongqing, Fujian, Guangdong, Guangxi, Guizhou, Hubei, Hunan, Jiangxi, Sichuan, Yunnan	Sichuan (Mao Xian)	Hubei (Fang Xian, Badong)	Hubei (Zhuxi)

## Supplementary Material

XML Treatment for Deutzia setchuenensis

XML Treatment for Deutzia setchuenensis var. longidentata

XML Treatment for Deutzia setchuenensis Franch. var. macrocarpa
